# Treatment of Coking Wastewater Using Hydrodynamic Cavitation Coupled with Fenton Oxidation Process

**DOI:** 10.3390/molecules29051057

**Published:** 2024-02-28

**Authors:** Dongmei Deng, Ting Huang, Qing Li, Yongchun Huang, Yufei Sun, Jieliang Liang, Jintian Li

**Affiliations:** 1 Guangxi Key Laboratory of Green Processing of Sugar Resources, College of Biological and Chemical Engineering, Guangxi University of Science and Technology, Liuzhou 545006, China; deng-dongmei@163.com (D.D.); m18894898963_1@163.com (T.H.); lq1468371790@126.com (Q.L.); huangyc@gxust.edu.cn (Y.H.); 2 Institute of Ecological Science, Guangzhou Key Laboratory of Subtropical Biodiversity and Biomonitoring, Guangdong Provincial Key Laboratory of Biotechnology for Plant Development, School of Life Sciences, South China Normal University, Guangzhou 510631, China; lijintian@m.scnu.edu.cn

**Keywords:** coking wastewater, imping stream–hydrodynamic cavitation, Fenton

## Abstract

Effective and economical processes for the advanced treatment of coking wastewater were urgently needed to reduce the persistent organic pollutants of external drainage. In the present work, we investigated the degradation of organic pollutants in coking wastewater through IHC/FO (imping stream hydrodynamic cavitation (IHC) coupled with the Fenton oxidation (FO) process) and IHC alone for their feasibility in the advanced treatment of coking wastewater. To select the optimum parameters, attention was paid to the effects of main operation conditions including inlet fluid pressure, medium temperature, initial pH, reaction time, and initial Fe(II) and initial H_2_O_2_ concentrations. The results showed that the effects of conditions that need energy to be maintained (such as initial pH and inlet pressure) on the organic pollutant removal efficiency through IHC/FO were less pronounced than those through IHC alone. Moreover, the application of IHC/FO could remove more organic pollutants from coking wastewater than IHC even at an energy-efficient condition. For example, the highest COD removal efficiency of 12.5% was achieved in the IHC treatment at 0.4 MPa, pH 3, and 60 min for the reaction time. In the case of IHC/FO, the maximum COD removal of 33.2% was obtained at pH 7, 0.1 MPa, 12 mmol/L H_2_O_2_, and 3 mmol/L Fe^2+^ after reacting for 15 min. The ultraviolet and visible spectrophotometry (UV-Vis) absorption spectra and gas chromatography and mass spectrometry (GC–MS) analysis further revealed that the kinds and amounts of pollutants (especially those that had benzenes) remaining in water treated through IHC/FO were much fewer and smaller than in water treated through IHC alone. The better performances of IHC/FO than IHC alone were likely related to the more hydroxyl radicals produced through IHC/FO. Taken together, our findings indicate that IHC/FO has great application potential in the advanced treatment of coking wastewater.

## 1. Introduction

Coking wastewater has been produced in the coal coking processes (coke making, condensation, and coke quenching), coal gas purification, and coking by-product recovery processes [1,2]. Varieties of inorganic and organic pollutants have been identified in coking wastewater, among which ammonium, sulfate, phenolic compounds, benzene, and thiocyanate are of high toxicity [3,4]. The most widely used processes for coking wastewater treatment include caustic treatment and steam stripping to reduce the contaminant load, followed by conventional biological treatment, commonly known as the activated sludge process [5]. Due to the presence of many refractory and toxic compounds, the activated sludge process is not efficient for the removal of organic pollutants [6]. Consequentially, it is difficult for these effluents to meet increasingly stringent effluent discharge limits and to be recycled for many beneficial reuse purposes [7]. Moreover, the toxicological properties of these effluents mean that they must be further treated prior to their discharge to the environment. Many studies are now focused on the application of advanced oxidation processes (AOPs) in the advanced treatment of coking wastewater, studying processes such as ozone (O_3_), hydrogen peroxide (H_2_O_2_), and Fenton oxidation (FO) [8,9]. In an AOP, oxidants generate reactive oxygen species (ROS) such as hydroxyl radicals (OH), with an oxidation potential (E_0_) of 2.8 eV, that are non-selective species with high oxidation potentials [10]. However, most of these studies are still in the laboratory stage and have not been applied in practice due to the complexity and difficult degradation of coking wastewater. Therefore, efforts on advanced processes for coking wastewater treatment are still necessary. Moreover, the higher costs of chemicals make using an AOP an economically infeasible approach, driving research into developing effective combinations [10].

Cavitation is a process of cavity generation, growth, and final implosion resulting in the generation of chemical and physical effects including the formation of hotspots, turbulence, and some highly reactive free radicals such as HO, H, HOO, HO_2_, and H_2_O_2_ [11]. The chemical effects of cavitation ensure that generated radicals (including some from the pollutants based on dissociation) further react with compounds, causing their mineralization while the physical effects such as turbulence and micro-streaming help mainly in terms of the enhanced consumption of the oxidants based on the elimination of mass transfer effects [11]. In hydrodynamic cavitation (HC), cavitation generation is based on the local variations in the pressure and kinetic energy in the flowing liquid due to geometric constrictions such as nozzles, venturi, or orifices [12,13]. HC has been used in wastewater treatment in recent years and can successfully degrade various aromatic compounds [14,15,16]. Although HC has shown good applicability for wastewater treatment, the complete mineralization of complex pollutants using HC alone is challenging due to an insufficient oxidation capacity [10]. Hence, with an objective to increase the treatment efficiency, the combined treatment of HC with other oxidants (such as ozone, Fenton’s reagent, hydrogen peroxide, etc.) has been investigated [11,17]. It has been confirmed that the combination of HC and other oxidants is energy-efficient and cost-effective compared to individual techniques for the treatment of single pollutants in wastewater such as p-nitrophenol, Orange G, and oxidant benzene [18,19]. The intensification of treatment can be achieved with cavitation effects, giving an enhanced generation of reactive free radicals and better utilization of added chemicals based on increased mass transfer [11,17,18,20]. On the one hand, the addition of oxidants such as H_2_O_2_ provides supplementary oxidizing agents. On the other hand, HC enhances the decomposing of oxidants and also the micro mixing of reactants. Thus, the combination of HC and AOPs could have a better effect as compared to the singular application of these processes.

The combination of HC and other AOPs also could effectively remove organic pollutants such as dye, cork, and pharmaceutical wastewater in real wastewater treatment [20,21,22]. For example, the use of HC in combination with FO as a pre-treatment for the conventional biological treatment of pharmaceutical wastewater could remove more than 50% COD while the individual operation of HC resulted in only 21.6% COD removal efficiency. However, not many literature reports are available based on the treatment of an effluent obtained directly from any industry using the combined actual operation of HC and oxidants due to the complex composition of wastewater. In particular, the detailed pollutant change in real wastewater during the treatment has not been well studied. Moreover, the cost of using HC or HC combined with oxidizers to treat actual wastewater also has not been extensively studied. To some degree, the absence of these studies limits the practical application of HC combined with AOPs.

To our knowledge, little work has addressed the advanced treatment of coking wastewater via HC or HC coupling with FO, highlighting the novelty of the current work. Considering the physical and chemical effects induced by the bubble collapse in HC, we supposed that HC and HC/FO both had application potential in coking wastewater advanced treatment. Moreover, HC/FO was assumed to be more efficient in treating coking wastewater due to the higher production of quantum free radicals. Therefore, in the present work, we investigated imping stream hydrodynamic cavitation (IHC) coupled with Fenton oxidation to treat real coking wastewater following biological treatment. The impinging stream (IS) is another novel chemical-intensive technology, and IHC is supposed to obtain an improved degradation efficiency of organic macromolecules [23]. Thus, the main goal of this work was to investigates the feasibility of using HC and HC/FO processes to treat biologically treated coking wastewater with laboratory-level IHC equipment. In detail, this research was conducted to (1) evaluate the effectiveness of IHC, FO, and IHC/FO for treating a biotreated coking plant effluent; (2) analyze the effects of operating process parameters of HC and HC/FO on the removal efficiency of organic pollutants in coking wastewater and provide guidelines; (3) analyze and recommend an efficient and economical degradation process by calculating the cost of removal of COD in coking wastewater through IHC or IHC/FO; and (4) offer an insight into the mechanism of coking wastewater degradation through IHC or IHC/FO through an analysis of the pollutant change in coking wastewater.

## 2. Results

### 2.1. Effect of Treating Time

**Figure 1:**
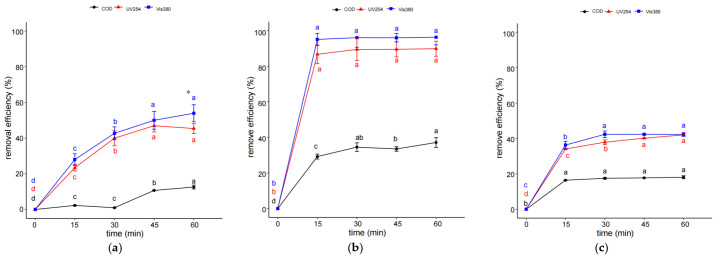
Effect of reaction time on organic pollutant removal efficiency through IHC (**a**), IHC/FO (**b**), and FO (**c**). Note: experiments in (**a**) were carried out at temperature of 40 °C, inlet pressure of 0.4 MPa, and pH 3; those in (**b**) were carried out at temperature of 40 °C, inlet pressure of 0.4 MPa, 12 mmol/L H_2_O_2_, 3 mmol/L Fe(II), and pH 3; and those in (**c**) were carried out at temperature of 40 °C, 12 mmol/L H_2_O_2_, 3 mmol/L Fe(II), and pH 3. * The different letters within the different treatments indicate significant differences (*p* < 0.05).

Figure 1a shows that the removal efficiencies of UV254, Vis380, and chemical oxygen demand (COD) in coking wastewater increased with the rise in reaction time and reached the maximum after 45 min or 60 min of IHC treatment. Moreover, the removal efficiencies of UV254, Vis380, and COD at 30 and 45 min were all significantly higher than those at 30 min and 15 min (*p* < 0.05). It was observed that increasing the reaction time of IHC/FO treatment could also improve the removal efficiency of organic pollutants in coking wastewater at first. As for FO, the removal efficiency of UV254 and COD reached the maximum after 30 min while the difference in terms of the removal efficiencies of the three organic pollutants among different reaction times was not so significant as those values in IHC treatment. The maximum removal efficiency of COD in FO was a little higher than that of IHC. In contrast, the removal efficiencies of UV254, Vis380, and COD reached the maximum after 15 min through IHC/FO treatment while the differences in terms of the removal efficiencies of the three organic pollutants among different reaction times were rather similar. Moreover, the maximum removal efficiency values of UV254, Vis380, and COD in IHC/FO treatment were 95%, 96%, and 37%, which were also obviously higher than those in IHC or FO treatment. There have been many studies on FO reaction conditions; thus, in subsequent experiments, IHC and IHC/FO treatments were further studied and the reaction times of IHC and IHC/FO were fixed at 60 min and 15 min, respectively.

### 2.2. Effect of pH

**Figure 2:**
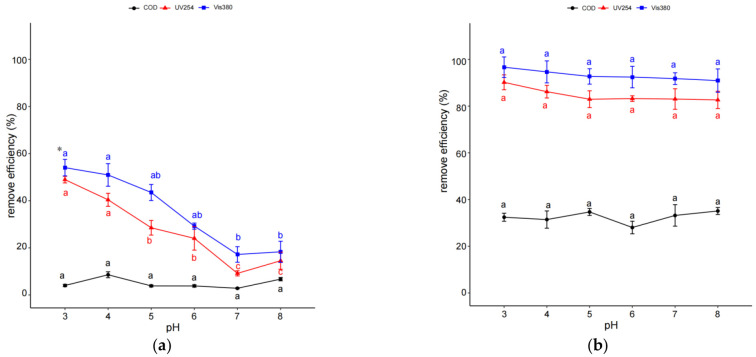
Effect of pH on organic pollutant removal efficiency through IHC (**a**) and IHC/FO (**b**). Note: experiments in (**a**) were carried out at temperature of 40 °C, inlet pressure of 0.4 MPa, and reaction time of 60 min; those in (**b**) were carried out at temperature of 40 °C, inlet pressure of 0.4 MPa, 12 mmol/L H_2_O_2_, 3 mmol/L Fe(II), and reaction time of 15 min. * The different letters within the different treatments indicate significant differences (*p* < 0.05).

The effects of initial pH on organic pollutant removal efficiency with treatments of IHC and IHC/FO were investigated and are shown in Figure 2. As shown in Figure 2, an increase in the pH of coking wastewater caused a sharper decrease in the removal efficiencies of UV254 and Vis380 through IHC than through IHC/FO. In IHC treatment, UV254 and Vis380 removal efficiency values decreased by more than 35%, and the pH varied from 4 to 7. The removal efficiency values of UV254 and Vis380 at pH 3 and 4 were significantly higher than those at pH 7 or 8 (*p* < 0.05). But UV254 and Vis380 removal efficiency in coking wastewater just decreased by about 5% during the same pH variation in IHC/FO treatment, and the differences in terms of removal efficiency between the three pollutants all did not reach a significant level (*p* > 0.05). Moreover, it was obvious that IHC/FO treatment could remove relatively more organic pollutants even with less reaction time than IHC treatment. Based on these results, the optimal pH value at 3 was selected in the following experiments using IHC treatments. pH 7 was considered a better choice for the following IHC/FO treatments in the view of practical application.

### 2.3. Effect of Temperature

**Figure 3:**
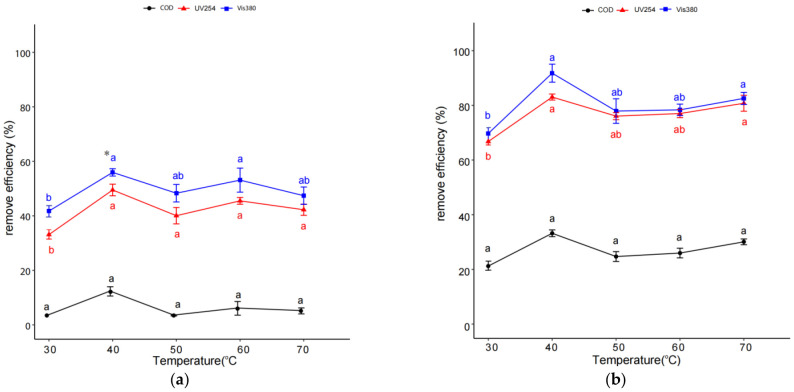
Effect of temperature on organic pollutant removal efficiency through IHC (**a**) and IHC/FO (**b**). Note: experiments in (**a**) were carried out at pH 3, inlet pressure of 0.4 MPa, and reaction time of 60 min; those in (**b**) were carried out at pH 5, inlet pressure of 0.4 MPa, 12 mmol/L H_2_O_2_, 3 mmol/L Fe(II), and reaction time of 15 min. * The different letters within the different treatments indicate significant differences (*p* < 0.05).

Figure 3 shows that the removal efficiency values of COD, UV254, and Vis380 in coking wastewater marginally increased as the operating temperature of the system increased from 30 °C to 40 °C in both IHC and IHC/FO treatment. However, the removal efficiency values of organic pollutants in coking wastewater showed some differences in terms of the further increase in the temperature until 70 °C. In IHC treatment, when temperature increased from 40 °C to 70 °C, the removal efficiency values of organic pollutants, especially UV254 and Vis380, were similar to those at 40 °C, though there was some small fluctuation in the removal efficiency values of organic pollutants (Figure 3a). By contrast, in IHC/FO treatment, a further increase in the temperature until 70 °C always gave lower rates of removement of organic pollutants in coking wastewater (Figure 3b) than those at 40 °C. Taken together, both IHC and IHC/FO treatments were carried out at 40 °C in the following experiments.

### 2.4. Effect of Inlet Pressure

**Figure 4:**
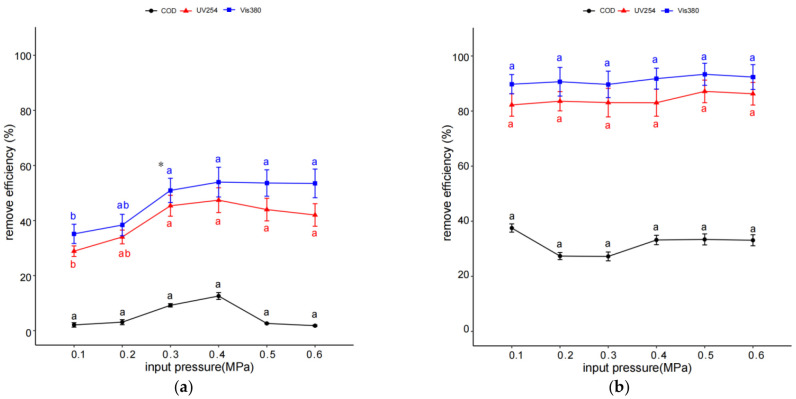
Effect of inlet pressure on organic pollutant removal efficiency through IHC (**a**) and IHC/FO (**b**). Note: experiments in (**a**) were carried out at pH 3, 40 °C, and reaction time of 60 min; those in (**b**) were carried out at pH 5, 40 °C, 12 mmol/L H_2_O_2_, 3 mmol/L Fe(II), and reaction time of 15 min. * The different letters within the different treatments indicate significant differences (*p* < 0.05).

It was observed that the quantities of organic pollutants in coking wastewater significantly decreased with an increase in the inlet pressure and reached a maximum (0.4 MPa) (*p* < 0.05) and then varied a little in higher pressure during IHC treatment (*p* > 0.05) (Figure 4a). In IHC treatment, the highest removal efficiency values of UV254, Vis380, and COD were 45%, 54%, and 12%, respectively (Figure 4a). Regarding IHC/FO, an increase in inlet pressure had little effect on the removal efficiency values of organic pollutants (*p* < 0.05). When inlet pressure increased from 0.1 to 0.5 MPa, the highest removal efficiency values of UV254, Vis380, and COD were 87%, 93%, and 37%, respectively, which all varied by less than 10% (Figure 4b). The inlet pressure values at 0.4 MPa and 0.1 MPa were selected as the optimal conditions in the following IHC and IHC/FO treatments.

### 2.5. Effects of H_2_O_2_ and Fe(II)

**Figure 5:**
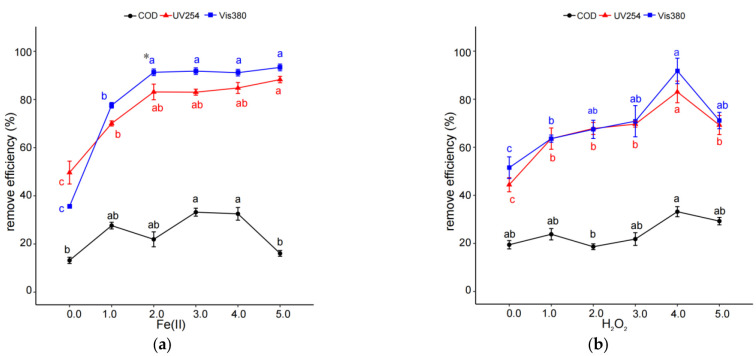
Effects of concentrations of Fe(II) (**a**) and H_2_O_2_ (**b**) on organic pollutant removal efficiency through IHC/FO. Note: experiments in (**a**) were carried out at pH 7, 40 °C, inlet pressure 0.1 MPa, reaction time of 15 min, and 12 mmol/L H_2_O_2_; those in (**b**) were carried out at pH 7, 40 °C, inlet pressure 0.1 MPa, reaction time of 15 min, and 3 mmol/L Fe(II). * The different letters within the different treatments indicate significant differences (*p* < 0.05).

With an increase in Fe (II) concentration from 0 mmol/L to 3 mmol/L, the removal efficiency values of COD, UV254, and Vis380 significantly increased (*p* < 0.05). With a further increase in Fe (II) concentration to 5.0 mmol/L, the extents of UV254 and Vis380 reduction became similar to those at 3 mmol/L Fe (*p* > 0.05) while the extents of COD reduction were significantly reduced (*p* < 0.05) (Figure 5a). To investigate the effect of H_2_O_2_ on the removal of organic pollutants in coking wastewater through IHC/FO treatment, experiments were carried out for different H_2_O_2_ concentrations ranging from 0 to 15 mmol/L. Figure 5b showed that increasing the H_2_O_2_ concentration could increase the removal efficiency values of organic pollutants at the condition of 0 to 12 mmol/L H_2_O_2_, and the maximum removal rate was reached at 12 mmol/L.

### 2.6. Optimization of Reaction Conditions and the Effect of Interaction between Conditions

Design Expert software 13.0 was further used to design the RSM experiments and explore the effects of influential factors on the treatment results, especially the interaction between factors. For IHC, 17 experiments were run and the results obtained were used to analyze the effects of factor variables, namely pH (X_I1_), temperature (X_I2_), and reaction time (X_I3_), on the percent of COD removal (Y). In IHC/FO, a similar 17 experiments were carried out involving the following variables: X_IF1_ (dose of FeSO_4)_, X_IF2_ (dose of H_2_O_2_), and X_IF3_ (pH). The complete design matrix, as well as the results of COD removal efficiency for IHC and IHC/FO, is presented in Table 1 and Table 2.

A regression model was used to establish first- or second-order polynomial equations between factor variables and the percent of COD removal. The data from IHC and IHC/FO were fitted to two models, presented as Equations (1) and (2), respectively.

COD removal (%) = 10.86 − 1.57X_I1_ − 1.37X_I2_ − 0.21X_I3_ + 0.57X_I1_X_I2_ + 0.40X_I1_X_I3_ − 0.10X_I2_X_I3_ − 1.04X_I1_^2^ − 1.14X_I2_^2^ − 0.0675X_I3_^2^ (1)

COD removal (%) = 19.55 + 7.23X_IF1_ − 4.00X_IF2_ − 2.06X_IF3_ + 2.77X_IF1_X_IF2_ − 2.48 X_IF1_X_IF3_ − 1.37X_IF2_X_IF3_ (2)

The significance and adequacy of the model were tested via ANOVA (Table 3 and Table 4). The F-test of the regression models produced very low *p*-values (<0.0001), indicating that both models were of high significance. The determination coefficients (R^2^) of the models indicated that 98.50% and 94.89% of the total variability could be explained by the models for the IHC and IHC/FO, respectively. The values of the adjusted determination coefficient (adjusted R^2^ = 0.9658 and 0.9182) also proved the high significance of both models. In addition, the coefficients of variation (C.V. = 3.29% and 9.52%) were less than 10, suggesting the high precision and reliability of the experiments.

The significance levels of all regression coefficients are also given in Table 3 and Table 4. It can be seen that for the IHC, both the p of the initial pH (X_I1_) and temperature (X_I2_) as well as the interaction of the initial pH with temperature (X_I1_X_I2_) and of the initial pH with reaction time (X_I1_X_I3_) were less than 5%. The results indicated that initial pH, temperature, and their interaction, as well as the interaction between initial pH and reaction time, play significant roles in COD removal (Table 3). For the IHC/FO reactions, X_IF1_ (dose of FeSO_4)_, X_IF2_ (dose of H_2_O_2_), and X_IF3_ (pH) all play significant roles in COD removal, which was further confirmed by their p value (*p* < 0.05) (Table 4). Amongst the interacting factors, the interaction between FeSO_4_ and H_2_O_2_ (X_IF1_X_IF2_) and interaction between FeSO_4_ and pH (X_IF1_X_IF3_) were found to be having significant effects on COD removal in IHC/FO.

**Figure 6:**
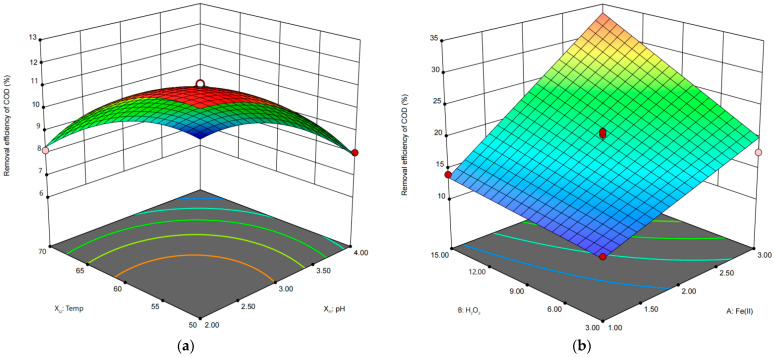
Three-dimensional response surface diagrams showing the most significant effects of interactions between pH (X_I1_) and temperature (X_I2_) in IHC treatment (**a**), as well as those between Fe(II) concentration (X_I1_) and H_2_O_2_ concentration in IHC/FO treatment (**b**), for COD removal (*p* < 0.05).

Figure 6a shows the 3D plot of the IHC treatment with the variables of pH and temperature, giving the significant linear and interaction effects on the process efficiency (*p* < 0.05). It was apparent that when the pH was reduced from 4 to 2 and the temperature was lowered from 70 °C to 50 °C, the COD removal rate gradually increased. However, when the pH was lowered to 3 or the temperature was lowered to 60 °C, there was no significant change in the UV254 removal rate with the variation of the reaction condition.

Figure 6b shows the 3D plot of the COD removal efficiency of the IHC/FO treatment regarding the interaction effects between Fe(II) concentration and pH, of which the linear or interaction coefficients were significant (Table 4). It could be found that COD removal increased gradually when the pH was decreased from 7 to 5 and the Fe(II) concentration was increased from 1 mol/L to 3 mmol/L.

### 2.7. GC–MS Analysis

Gas chromatography–mass spectrometry (GC–MS) was further used to analyze the change in the organic compound composition of the coking wastewater after IHC or IHC/FO treatment. It was shown that there were fourteen peaks in the raw wastewaters while only six and four peaks remained in the wastewater samples treated using IHC and IHC/FO, respectively (Figure S1, Supplementary Materials). The identified compounds are listed in detail in Table S1 (Supplementary Materials).

The results showed that more kinds of organic pollutants were identified in coking wastewater, and the dominant organics were benzene-series compounds like 1,3-dimethylbenzene, 1,4-dimethyl-benzene, and ethylbenzene. A few long-chain compounds such as n-dodecane and n-tridecane were also found in coking wastewater. After IHC treatments, long-chain compounds and many benzene-series compounds (including benzene, 1,2-dichloropropane, cumene, 2-ethyl-2-hexenal, and benzaldehyde) disappeared from the treated wastewater. Consistent with this, the UV-Vis spectra showed that there were obvious strong peaks over the range of 200–240 nm and relative high absorption over the range of 300–375 nm in raw coking wastewater (Figure S2, Supplementary Materials).

Meanwhile, the concentrations of some benzene-series compounds such as ethylbenzene, 1,3-dimethylbenzene, and 1,4-dimethyl-benzene were significantly decreased. The above-mentioned pollutants in coking wastewater removed through IHC treatment could also be effectively removed through IHC/FO treatment. Moreover, 1,4-dimethyl-benzene and ethylbenzene were totally removed through IHC/FO treatment. However, GC–MS analysis also revealed that relatively high concentration of toluene still remained in coking wastewater even after either IHC or IHC/FO treatment (Figure S1, Table S1, Supplementary Materials). In agreement with the results of the GC–MS, it was found in the UV-Vis spectra that the contaminants that had absorption peaks between 300 and 375 nm were well removed through IHC and IHC/FO. Meanwhile, more pollutants whose absorption peaks lay in the range of 200–240 nm were removed through IHC/FO treatment than through IHC treatment (Figure S1, Supplementary Materials).

### 2.8. Operating Cost

A comparison of the IHC, IHC/FO, and FO treatment costs was conducted, as shown in Table 5. To remove 1 g COD from coking wastewater, the cost of IHC/FO treatment ranged from 0.014 CNY/g COD to 0.016 CNY/g COD, which was approximately one-fifth to one-third less than the corresponding costs of the IHC and FO treatment, respectively. More important, the IHC/FO treatment could achieve a higher degree of COD removal than the IHC and FO treatments.

## 3. Discussion

The treatment of coking wastewater has been a big challenge because a considerable quantity of unknown organic compounds still remains in the biologically treated effluents, requiring further treatment. As one of the AOPs, Fenton utilizes the strong oxidation potential of the free radical and the coagulation from the formation of ferric hydroxide to oxidize and remove pollutants, which has been well studied in treating coking wastewater [24,25,26]. Meanwhile, the combination of IHC and FO processes for the treatment of coking wastewater has rarely been reported. It has been revealed that the combination of HC and AOPs is highly energy-efficient as compared to individual techniques to treat the wastewater for a variety of pollutants such as p-nitrophenol [20], Orange G [19], phenolic derivatives [19], and pesticide [27]. Consistent with these results, our study showed that the removal efficiencies of UV254, Vis380, and COD in coking wastewater through IHC/FO treatment were distinctly higher than not only those through IHC or FO alone but also the sum of those through IHC or FO alone at similar conditions (including pH, temperature, and inlet pressure) in a shorter time (Figure 1). This phenomenon could be attributed to more available amounts for hydroxyl radical generation due to the added H_2_O_2_ [28,29]. The addition of a Fenton catalyst such as Fe(II) into the HC–H_2_O_2_ system leads to further usually significant enhancement [30] based on the series of reactions involving the formation of a complex and breakage of the same into Fe(II) ions under the cavitational effects [31]. Moreover, HC also prevents the agglomeration of the ferrous ions, providing a better contact with the contaminants present in wastewater [32]. Consistent with our findings, combining HC and FO processes can increase COD removal by more than 20% in both cork and pharmaceutical wastewater compared to HC alone [20]. This demonstrates the advantages of this approach in treating real industrial wastewater. Furthermore, while coking wastewater and other industrial wastewaters share the characteristic of containing difficult-to-biodegrade organic compounds, their specific pollutants differ. This suggests that the treatment of challenging industrial wastewaters using HC/FO is applicable across a wide range of water types.

The development of a cost-effective technology for wastewater treatment is very important for achieving successful operations for the treatment of wastewater in actual industrial-scale operations [31]. The pH, temperature, and inlet pressure are important operating parameters of the HC system, and Fe(II) and H_2_O_2_ are another two important operating parameters of FO [33,34,35]. The optimization of these operating parameters could maximize the effectiveness of IHC or IHC/FO treatment and reduce operating costs to a certain extent. It could be observed that initial pH and inlet pressure had more effects on the organic pollutant removal efficiencies through IHC than through IHC/FO (Figure 1). The result of RSM also showed that pH was the most important factor that influenced the removal of COD during IHC treatment (Table 3). The HC process was favored at low pH conditions, which can be attributed to the fact that hydroxyl radicals are favored to be generated under acidic conditions in HC and that the oxidation capacity of hydroxyl radicals is also higher under acidic conditions [36,37]. The higher removal efficiency of organic pollutants in coking wastewater with an increasing pressure up to 0.4 MPa can be attributed to the enhancement of the hydroxyl radical production as a result of the intensification of cavitation activity [38,39]. By contrast, distinctively more organic pollutants could be removed at neutral (pH 7) and relatively low inlet pressures (0.1 MPa) as the removal of organic pollutants was not sensitive to pH and inlet pressure in IHC/FO treatment in this study. The result of RSM also showed that pH was not the most important factor that influenced the removal of COD during IHC treatment (Table 4). Korpe also found that pH was not having a major impact on COD removal efficiency in tannery wastewater when using HC coupled with AOPs [32]. This might be due to the enhancement of the hydroxyl radical and cavitation number production with the help of H_2_O_2_ and Fe(II) [36,40].

Different from pH and inlet pressure, a more distinct optimum temperature (40 °C) and Fe(II) (3 mmol/L) and H_2_O_2_ (12 mmol/L) levels were determined during IHC/FO treatment in a one-way experiment. In IHC/FO, as the temperature of the liquid increased, its vapor pressure increased substantially, which could generate higher amounts of free radicals. On the other side, at higher operating temperatures, an increase in the vapor content in the bubble decreased the collapse pressure, resulting in a net decrease in the energy being released at the collapse and thus reducing the generation of reactive hydroxyl radicals in the system [41,42]. Moreover, at higher temperatures, the activity of the Fenton process was retarded due to the thermal decomposition of H_2_O_2_ [26], which also reduced the concentration of reactive oxygen species to some extent. A high concentration of Fe(II) leads to an increase in process efficiency for the classic Fenton reaction because Fe(II) determines the rate of the conversion of H_2_O_2_ to free radicals (OH) [25]. The RSM showed that Fe(II) concentration was also the most important factor affecting the removal of organic matter through the IHC/FO process, which might be for the same reason. Meanwhile, the observed optimal Fe(II) and H_2_O_2_ can be explained based on the fact that beyond the optimum quanta of Fe (II) ions or H_2_O_2_, the excess ions scavenge the hydroxyl radicals, reducing the availability for pollutants, which ultimately decreases the reduction in organic pollutants [26,28,41,43]. Similar results have been reported for the degradation of methyl parathion and dichlorvos using HC/FO [44,45]. Moreover, considering that the COD determination process is easily disturbed and produces contamination, TOC may be considered as a substitute for COD in the future to better characterize the treatment effect of IHC or IHC/FO on wastewater [46].

In IHC, the interaction of pH and temperature had a significant effect on the removal of COD. A higher temperature might have increased the ionization of water and favored the generation of ·OH radicals, and also offered a higher oxidation potential and a lower rate of recombination of ·OH radicals [46,47]. As for IHC/FO, the interaction of Fe(II) and H_2_O_2_ concentration also influenced the COD removal significantly (*p* < 0.05). In addition, it should be noted that the RSM modeling could not give information on reaction mechanisms [25]. To achieve an optimal treatment effect, further research with new analytical methods is needed to explore the mechanisms acting on the removal of pollutants in wastewater when treated using IHC and IHC/FO. For example, electron paramagnetic resonance (EPR) and other radical scavenging analyses, which could identify the active species of the generated reactive oxygen species, could confirm the existence and contribution of hydroxyl radicals to the removal of pollutants [48]. Moreover, three-dimensional excitation–emission matrix spectroscopy (3D-EEM) analysis could reflect the degradation of complex water bodies to a certain extent, which might help us understand the influence of reaction factors such as residual H_2_O_2_ on the COD removal rate and could give us important clues to understand why IHC/FO failed to achieve a high COD removal rate for the coking wastewater in this study. The further development of these analyses could help us gain a deeper understanding the mechanism to remove pollutants in coking wastewater through IHC or IHC/FO.

In brief, the optimum experimental conditions of IHC/FO in a one-way experiment have been identified as being at 40 °C, with a cycle time of 15 min, pH of 7, inlet pressure of 0.1 MPa, H_2_O_2_ concentration of 12 mmol/L, and Fe(II) concentration of 3 mmol/L. Under the conditions, the removal efficiencies of UV254, Vis380, and COD reached 86%, 96%, and 33%, respectively. It is worth mentioning that if the IHC/FO was applied to the advanced treatment of real coking wastewater under these conditions, no additional pH adjustment and temperature compensation are needed. Based on power and reagent requirements, the costs required for the approach of using IHC/FO under optimal and similar reaction conditions in this study were notably lower than those for IHC or FO (Table 5). It is important to note that when the IHC/FO process is used for the advanced treatment of coking wastewater on an industrial scale, only a small number of IHC devices need to be added on the basis of the coagulation tank, and the cost of renovation and equipment investment would be rather low. Thus, IHC/FO might be an economical and effective advanced treatment option in the future. Though the estimation of IHC/FO operating costs was also lower than that of FO reported, it was a little higher than that of a synchronized oxidation–adsorption (SOA) technology in the advanced treatment of coking wastewater [49]. Thus, it is also the need of the hour to further reduce the costs of IHC/FO in large-scale applications without compromising effectiveness through the optimization of reaction conditions and improvement of FO reagent types. Moreover, the estimation of IHC/FO operating costs in this study was not precise enough due to the limitation of treatment size. Scale-up experiments are needed to advance the application of the IHC/FO process.

It was obvious that the removal efficiency rates of UV254 and Vis380 were greater than the COD at the same environmental condition in either IHC or IHC/FO treatment, which indicates that the oxidation process through IHC and IHC/FO might just break the chemical bond that organic pollutants form with coking wastewater and produce some small organic molecules. The further UV-Vis spectra and GC–MS analysis of coking wastewater after IHC or IHC/FO treatment confirmed it. The overall absorbance of the curve, especially for benzenes such as polycyclic aromatic hydrocarbon and nitrogen heterocycles with absorption between 300 and 375 nm, decreased significantly after IHC/FO or IHC treatment, which indicated that both of the two treatments could help the oxidation of these compounds [50,51]. GC–MS analysis further indicated that these polycyclic aromatic hydrocarbon and nitrogen heterocycles included 2-ethyl-2-hexenal, n-undecane, n-dodecane, and n-tridecane. It is well known that polycyclic aromatic hydrocarbon and nitrogen heterocycles might be harmful to organisms [52]. A reduction in these compounds could effectively reduce the possible toxicity of coking wastewater. Moreover, the results also indicated IHC/FO treatment could remove more low-molecular-weight pollutants such as 1,4-dimethyl-benzene and ethylbenzene, with strong absorption peaks between 200 and 240 nm, which might due to the larger amounts available for hydroxyl radicals generated during IHC/FO treatment. Moreover, as shown in Table S1 (Supplementary Materials), 2-iodobutane, chlorobenzene, and 1,2-dichloropropane in the reactant have a chlorine compound or iodine compound. The attack of ROS, such as ·OH radicals, is one of the governing mechanisms of IHC and IHC/FO. During the treatment of IHC and IHC/FO, ·OH and H· radicals are released by the dissociation of trapped water molecules, then ·OH radicals diffuse into liquid and react with refractory organics, which might lead to the rearrangement of chemical bonds containing chlorine and iodine. Thus, a chloride compound appears in the effluent. Moreover, the relatively high concentration of toluene found in coking wastewater after either IHC or IHC/FO treatment meant that either IHC or IHC/FO treatment could not completely mineralize the organic pollutants in coking wastewater to CO_2_ and H_2_O and a part of toluene was produced from the degradation of other organic pollutants in coking wastewater. Toluene is one of the most commonly observed contaminants [53]. In addition, the effluent after IHC or IHC/FO treatment also contained a number of other organic pollutants, all of which, together with toluene, may have some biotoxicity. To ensure environmental safety, although these organic pollutants are not included in the China Coking Chemical Industry Pollutant Emission Standards (GB 16171-2012) [54], the biotoxicity of coking wastewater after IHC or IHC/FO deep treatment is recommended for further study.

In the future, in order to practically use IHC/FO in the advanced treatment of coking wastewater, in addition to further scaling up of the IHC/FO process, it is also necessary to consider its use in conjunction with other processes to further remove COD and to reduce the generation of toxic secondary small-molecule organic products.

## 4. Materials and Methods

### 4.1. Materials

Hydrogen peroxide (H_2_O_2_) (30%, *w*/*v*), ferrous sulphate heptahydrate (FeSO_4_·7H_2_O), potassium dichromate (KCr_2_O_3_), potassium hydrogen phthalate (C_8_H_5_KO_4_), silver sulfate (Ag_2_SO_4_), mercury sulfate (HgSO_4_), and other chemical reagents used in experiment were analytical-grade and purchased from Yijia Chemical and Apparatus Company (Liuzhou, China). Among them, H_2_O_2_ and FeSO_4_·7H_2_O was used as Fenton reaction reagents while KCr_2_O_3_, C_8_H_5_KO_4_, Ag_2_SO_4_, and HgSO_4_ all were used in the determination of COD. Solutions of sulfuric acid (H_2_SO_4_) (10%, *v*/*v*) and sodium hydroxide (NaOH) (0.1 N) were used for adjustment of pH. Double-distilled water, which was prepared currently in the laboratory using a double-distilled water plant, was applied to prepare solutions.

### 4.2. Characteristics of the Coking Wastewater

The coking wastewater was collected from the effluents of a coking wastewater treatment plant after Anoxic/Oxic (A/O) process in Liuzhou, China. The temperature, COD, pH, and UV254 and Vis380 levels in the coking wastewater were found to be 36–40 °C, 7.5–8.1, 235–275 mg L^−1^, 2.6–3.0, and 0.50–0.58.

### 4.3. Experimental Setup

**Figure 7:**
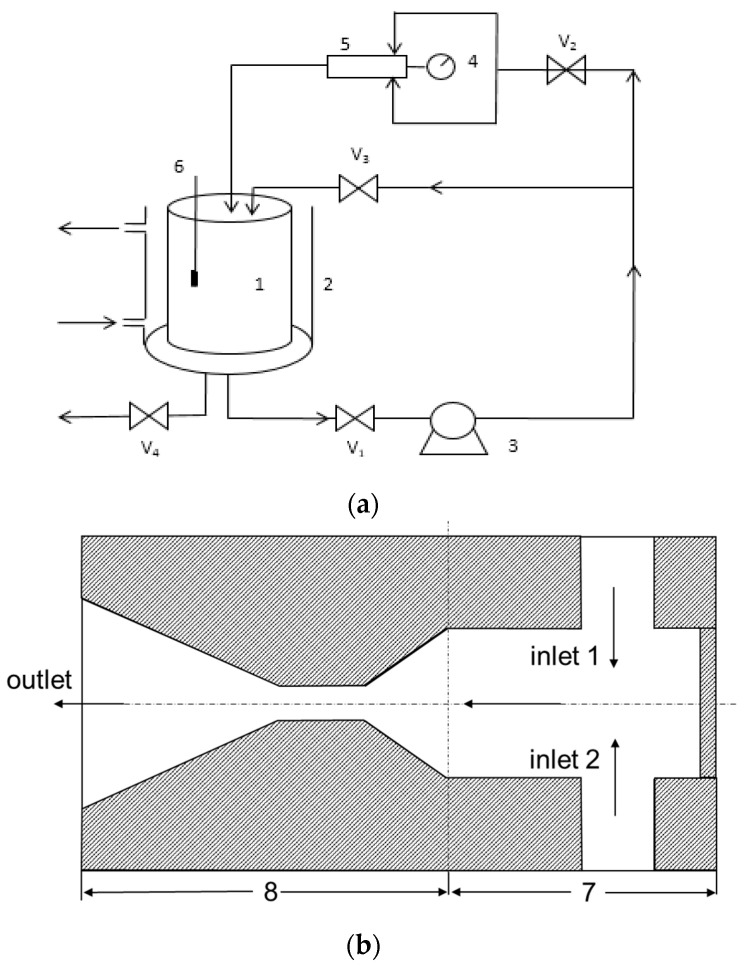
Schematic representation of the hydrodynamic cavitation setup (**a**) and structure of cavitation reactor (**b**). Note: 1—Storage tank; 2—circulation tank; 3—pump; 4—pressure gauge; 5—hytrodynamic cavitation reactor; 6—temperature meter; 7—imping stream; 8—venture tube; V1, V2, V3, V4—control valves.

The experimental setup for IHC and IHC/FO experiment is shown in Figure 7. The setup includes a holding tank (5 L), a positive displacement pump (power rating 1.1 kw), control valves (V1–V3), and cavitation reactor. The suction side of the pump is connected to the bottom of the tank and discharge from the pump branches through two lines: the main line and a bypass line (Figure 7a). The main line is divided into two branches before the cavitation reactor. The two branches impinge each other in the cavitation reactor and generate hydrodynamic cavitation with the assistance of venturi structure in cavitation reactor. The details of the structure of venture tube and imping stream in cavitation reactor are shown in Figure 7b. The bypass line is provided to control the liquid flow through the main line and obtain the expected inlet pressure. Both the mainline and bypass line terminate well inside the tank below the liquid level to avoid any induction of air into the liquid. The storage tank was filled with 3.5 L of coking wastewater. pH was then adjusted to the test level using NaOH (0.1 N) or H_2_SO_4_ (10%, *v*/*v*) solution before pump was started. For the combination involving Fenton, the required amount of Fe(II) and H_2_O_2_ was added to the holding tank in IHC/FO treatment with simultaneous stirring in the form of FeSO_4_·7H_2_O solid and 30% H_2_O_2_ solution after pH adjustment.

FO experiments were carried out in 0.5 L capacity reactor. First, 0.3 L coking wastewater was added to the reactor. Then, pH adjustment and reagent addition were the same as in the IHO/FO operation. Moreover, the contents were well mixed on a magnetic stirrer during FO treatments.

### 4.4. Comparison of IHC, IHC/FO, and FO at Different Treatment Times

Initially, IHC alone, FO alone, and IHC/FO were operated from 15 min to 60 min to optimize the reaction time and investigate the feasibility of the IHC and IHC/FO processes for treating coking wastewater.

During the IHC and IHC/FO treatments, the inlet pressure and temperature were kept constant at 0.4 MPa and 40 °C, respectively; FO reaction was only carried out at 40 °C. Meanwhile, initial pH was kept at 3.0 ± 0.1 for IHC treatment, IHC/FO treatment, and FO treatments. During FO treatments and IHC/FO treatments, the initial concentrations of FeSO_4_ and H_2_O_2_ for FO reaction were 3 mmol/L and 12 mmol/L. Three replications were performed for each treatment in this study.

There have been many studies on FO reaction conditions, so the further studies only concentrated on the optimization of the reaction conditions for IHC and IHC/FO. The optimum reaction times of both IHC alone as well as IHC/FO were kept in the residue part of experiments. The rest part of experiments were performed at the optimum reaction time of IHC/FO or IHC alone.

### 4.5. One-Way Experiments to Optimize Reaction Conditions for IHC and IHC/FO

To further maximize the extent of organic pollutant removal efficiency, one-way experiments were conducted to optimize other main operation conditions including inlet fluid pressure, medium temperature, initial pH, initial Fe(II), and initial H_2_O_2_ concentration for IHC and/or IHC/FO. In detail, the initial pH of solution was changed from 3.0 to 8.0, then the initial temperature of solution was changed from 30 °C to 70 °C and the inlet pressure was discussed by changing the inlet pressure from 0.1 MPa to 0.5 MPa during the treatment of IHC/FO or IHC alone. Moreover, the effects of H_2_O_2_ (0–15 mmol/L) and Fe(II) (0–5 mmol/L) on the degradation of coking wastewater using IHC/FO were also studied. The inlet pressure was adjusted using control valves. The pH was adjusted by adding H_2_SO_4_ or NaOH. The different experiments were performed under isothermal conditions, which were maintained by circulating cooling water through the surrounding water jacket. Thus, the temperature was practically constant, i.e., temperature fluctuation did not exceed beyond 0.5–1 °C. Three replications were performed for each treatment in this study.

### 4.6. Response Surface Methodology (RSM) of IHC and IHC/FO

RSMs are commonly used to study the effect of influential parameters on the treatment results [55]. In this study, RSM was employed to study the interaction effect of influential parameters and evaluate optimum conditions for the removal of UV254 in IHC and IHC/FO treatments. A three-factor three-level central composite design (CCD) was employed for optimization of factors. Doses of FeSO_4_ (X_IF1_), H_2_O_2_ (X_IF2_), and pH (X_IF3_) were tested factors for IHC/FO while pH (X_I1_), temperature (X_I2_) and reaction time (X_I3_) were those for IHC. Table 1 provides the range of independent tested factor variables (X_IF1_, X_IF2_, and X_IF3_) for IHC/FO and those (X_I1_, X_I2_, and X_I3_) for IHC. Each CCD consisted of 17 runs. Upper and lower limits of these independent variables were selected on the basis of one-way experiments. In order to reduce systemic bias, experiments were carried out in a random manner. During the IHC treatment, the inlet pressure was kept constant at 0.4 MPa. During the IHC/FO treatment, the inlet pressure, temperature, and reaction time were kept constant at 0.1 MPa, 40 °C, and 15 min.

### 4.7. Removal Efficiency of Coking Wastewater

The removal efficiency was defined as below:

removal efficiency (%) = 100% × (C_o_ − C_f_)/C_o_ (3)

Here, C_o_ represents the initial concentration of pollutants, and C_f_ is the concentration of pollutants after treatment. The removal efficiency of coking wastewater was determined using COD, UV254, aVis380. Among them, UV254 represented the unsaturated bond aromatic compounds and Vis380 reflected the content of color ingredients. COD was analyzed through a COD analyzer (DR890, HACH, Loveland, CO, USA). UV254 and Vis380 were represented as light absorbance of 3 mL sample measured with 1 cm quartz cell at 254 nm and 380 nm using UV-Vis spectrophotometer (UV2102, Unico, Shanghai Instrument, Shanghai, China), respectively [28].

### 4.8. UV-Vis and GC–MS Analysis

The organic composition of coking wastewater before and after IHC or IHC/FO treatment was first characterized using UV-Vis spectrophotometer (UV-2102, Unico, Shanghai Instrument, Shanghai, China) with a spectrometric quartz cell (1.0 cm path length); wavelength varied from 200 nm to 700 nm.

GC–MS was further used to identify the composition of coking wastewater before and after IHC or IHC/FO treatment. For GC–MS analysis, samples were extracted using CHCl_3_ into neutral, basic, and acid phases and then dried using the drying agent sodium sulfite and filtered using 0.45 μm syringe filters. The prepared samples were used for GC–MS analysis via an Agilent 7890A/5975C GC/MS analyzer equipped with a quartz capillary column with id of 0.25 mm, length of 30 m, and film thickness of 0.25 μm. The stationary phase was OV-101. Temperature for the gasification compartment was maintained at 280 °C. The temperature control program was followed by retaining at 50 °C for 1 min and then increasing to 280 °C with an increment of 15 °C min^−1^. The temperature for the MS ion source was 200 °C, and electron energy was 70 eV.

### 4.9. Analysis of One-Way Experiments

One-way experiments on factors including reaction time, inlet fluid pressure, medium temperature, initial pH, and initial Fe(II) and initial H_2_O_2_ concentrations were repeated three times. All experimental data were analyzed through ANOVA using SPSS 20.0 software (SPSS Inc., Chicago, IL, USA). Significant differences were tested using the Least Significant Difference (LSD) test at *p* < 0.05. Mean values and standard errors (SEs) were presented.

### 4.10. Analysis of RSM

The software Design Expert (version 13.0) was used to analyze the results and fit a model to express the empirical relationship in terms of coded factors between factor variables (X_I1_ (pH), X_I2_ (temperature) and X_I3_ (reaction time) for IHC; X_IF1_ (dose of FeSO_4)_, X_IF2_ (dose of H_2_O_2_), and X_IF3_ (pH) for IHC/FO) and response variable (Y (removal efficiency of COD)). The modeling was performed through adjustment of first- or second-order polynomial equations to the experimental responses. Analysis of variance (ANOVA) was used to evaluate the adequacy of the developed model with the determination coefficient (R^2^), adjusted determination coefficient (adjusted R^2^), and lack-of-fit test. The importance of model and identification of the main and interaction effects of variables were also estimated using ANOVA. The interaction between the process variables was illustrated by the two-dimensional (2-D) contour plots. Optimum conditions were predicted on the basis of p and F values. The optimum response variable for IHC and IHC/FO was then calculated using the fitted models and validated through the experiments.

### 4.11. Operating Cost

In this study, the electrical energy costs to maintain input pressure and material including FeSO_4_, H_2_O_2_, and H_2_SO_4_ to adjust pH were taken into account as major cost items in the calculation of the operating costs (OC) (CNY/g COD):

OC = aENC + bCC_FeSO4_ + cCC_H2O2_ + dCC_H2SO4_ (4)

Here, ENC is energy consumption to maintain input pressure (kWh/g COD). Meanwhile, CC_FeSO4_, cCC_H2O2_ and dCC_H2SO4_ represent chemical consumption of FeSO_4_, H_2_O_2_, and H_2_SO_4_ (g/g COD), respectively. Price provided in the China market in December 2023 for a was 0.072 CNY/kWh for electrical energy. The prices of b, c, and dare for FeSO_4_ (0.25 CNY/kg), 30% H_2_O_2_ (0.81 CNY/kg), and 98% H_2_SO_4_ (0.26 CNY/kg), respectively. Cost for electrical energy (kWh/g COD) was calculated in Equation (5) while the consumption of FeSO_4_, H_2_O_2_, and H_2_SO_4_ was calculated based on actual usage.

(5) $$ENC=\frac{P\times Q\times t}{v(C_{o(COD)}-C_{f(COD)})}$$

Here, P is inlet pressure (MPa), Q is flow rate of circulating wastewater (L/min), t is operating time (min), v is volume (L), C_o(COD)_ represents the initial concentration of COD, and C_f(COD)_ is the concentration of COD after treatment.

## 5. Conclusions

The degradation of organic pollutants in coking wastewater using hydrodynamic cavitation alone or combined with the Fenton oxidation process was investigated. The conclusions drawn from this study are summarized as follows:

(1) The combination of IHC with FO results in enhanced extents of degradation of organic pollutants in coking wastewater under similar environment conditions.
(2) The IHC is sensitive to solution pH, reaction time, and inlet pressure in coking wastewater treatment. An acidic condition, long-enough reaction time, and optimum inlet pressure and temperature are recommended for enhancing the extent of degradation through IHC.
(3) Initial pH, inlet pressure, and reaction temperature had smaller effects on the organic pollutant removal efficiency of IHC/FO than IHC alone. By contrast, more significant influences of Fe(II) or H_2_O_2_ on pollutant reduction in coking wastewater under IHC/FO treatment were investigated in this study.
(4) More kinds and more amounts of organic compounds in coking wastewater were removed through IHC/FO than through IHC, as determined through GC–MS analysis and UV-Vis spectra.

## Figures and Tables

**Table 1:** Process factors and their levels for IHC and IHC/FO in RSM.

Variables for IHC	Range and Level			Variables for IHC/FO	Range and Level		
	−1	0	1		−1	0	1
X_I1_: initial pH	2	3	4	X_IF1_: initial Fe(II) (mmol/L)	1	2	3
X_I2_: temperature (°C)	50	60	70	X_IF2_: initial H_2_O_2_ (mmol/L)	3	9	15
X_I3_: reaction time (min)	20	40	60	X_IF3_: initial pH	5	6	7

**Table 2:** Experimental layout designed by Design Expert and the corresponding experimental values of responses.

	Variables			Removal Efficiency of COD (%)	Variables			Removal Efficiency of COD (%)
	X_I1_	X_I2_	X_I3_		X_IF1_	X_IF2_	X_IF3_	
	Initial pH	Temperature (°C)	Reaction Time (min)		Initial Fe(II) (mmol/L)	Initial H_2_O_2_ (mmol/L)	Initial pH	
1	2	50	40	11.98	2	15	7	19.65
2	3	60	40	10.86	1	9	5	12.13
3	4	50	40	8.05	2	9	6	20.44
4	4	60	20	7.63	2	9	6	18.20
5	3	60	40	11.04	3	9	7	24.56
6	3	50	60	10.77	3	3	6	17.62
7	4	70	40	6.51	1	15	6	14.01
8	2	70	40	8.15	3	9	5	34.26
9	3	60	40	10.51	2	3	5	15.29
10	2	60	60	11.06	2	9	6	20.97
11	3	60	40	11.12	2	9	6	18.90
12	3	70	20	8.73	2	15	5	25.89
13	2	60	20	11.94	1	9	7	12.35
14	4	60	60	8.36	3	15	6	31.30
15	3	60	40	10.78	2	3	7	14.51
16	3	50	20	11.33	2	9	6	20.90
17	3	70	60	7.77	1	3	6	11.41

**Table 3:** The analysis of variance (ANOVA) of the regression models for COD removal (%) after IHC treatments.

Source in IHC	Sum of Squares	Df	Mean Square	F-Value	Prob > F
Model	47.98	9	5.33	51.52	<0.0001
X_I1_	19.78	1	19.78	191.2	<0.0001
X_I2_	15.04	1	15.04	145.39	<0.0001
X_I3_	0.3486	1	0.3486	3.37	0.109
X_I1_X_I2_	1.31	1	1.31	12.67	0.0092
X_I1_X_I3_	0.648	1	0.648	6.26	0.0508
X_I2_X_I3_	0.04	1	0.04	0.3866	0.5538
Residual	0.5004	3	0.1668	2.98	0.1594
Lack of fit	0.2238	4	0.0559		
Pure error	48.7	16			
Cor total	0.5004	3	0.1668	2.98	0.1594
R^2^	0.9850	Adjusted R^2^	0.9658	Adeq. Precision	23.86
C.V. (%)	3.29	Predicted R^2^	0.8289		

**Table 4:** The analysis of variance (ANOVA) of the regression models for UV254 removal (%) after IHC/FO treatments.

Source in IHC	Sum of Squares	Df	Mean Square	F-value	Prob > F
Model	643.12	6	107.19	30.95	<0.0001
X_IF1_	418.18	1	418.18	120.76	<0.0001
X_IF2_	128.16	1	128.16	37.01	0.0001
X_IF3_	34.03	1	34.03	9.83	0.0106
X_IF1_X_IF2_	30.69	1	30.69	8.86	0.0139
X_IF1_X_IF3_	24.6	1	24.6	6.1	0.0537
X_IF2_X_IF3_	7.45	1	7.45	2.15	0.1731
Residual	34.63	10	3.46		
Lack of fit	28.3	6	4.72	2.98	0.1548
Pure error	6.32	4	1.58		
Cor total	677.75	16			
R^2^	0.9489	Adjusted R^2^	0.9182	Adeq. Precision	18.81
C.V. (%)	9.52	Predicted R^2^	0.7781		

**Table 5:** Consumptions and costs of FO; different treatments of IHC and IHC/FO.

Treat	Time (min)	Condition				Removal Efficiency of COD(%)	Consumption				Costs (CNY/g COD)				
		Input Pressure (MPa)	pH	H_2_O_2_ (mmol/L)	FeSO_4_ (mmol/L)		ENC (kwh)	H_2_O_2_ (g/g COD)	FeSO_4_ (g/g COD)	H_2_SO_4_ (g/g COD)	H_2_SO_4_	ENC	H_2_O_2_	FeSO_4_	Total
FO	15	0.01	3.00	12.00	3.00	18.41	0.000	8.865	9.908	0.222	0.000	0.000	0.024	0.002	0.026
	30	0.01	3.00	12.00	3.00	19.49	0.001	8.374	9.359	0.222	0.000	0.000	0.023	0.002	0.025
	45	0.01	3.00	12.00	3.00	19.72	0.001	8.276	9.249	0.222	0.000	0.000	0.022	0.002	0.024
	60	0.01	3.00	12.00	3.00	20.10	0.002	8.119	9.075	0.222	0.000	0.000	0.022	0.002	0.024
IHC	45	0.50	3.00	0.00	0.00	10.67	0.024	0.000	0.000	0.222	0.000	0.024	0.000	0.000	0.024
	60	0.50	3.00	0.00	0.00	12.57	0.027	0.000	0.000	0.222	0.000	0.027	0.000	0.000	0.027
	60	0.40	3.00	0.00	0.00	12.57	0.022	0.000	0.000	0.222	0.000	0.022	0.000	0.000	0.022
IHC/FO	15	0.10	3.00	12.00	3.00	32.43	0.001	5.032	5.624	0.222	0.000	0.001	0.014	0.001	0.016
	15	0.10	4.00	12.00	3.00	31.43	0.001	5.192	5.803	0.020	0.000	0.001	0.014	0.001	0.016
	15	0.10	5.00	12.00	3.00	34.69	0.000	4.705	5.258	0.000	0.000	0.000	0.013	0.001	0.015
	15	0.10	7.00	12.00	3.00	33.20	0.001	4.916	5.494	0.000	0.000	0.001	0.013	0.001	0.015
	15	0.10	8.00	12.00	3.00	35.10	0.000	4.650	5.197	0.000	0.000	0.000	0.013	0.001	0.014
	15	0.10	7.00	12.00	3.00	33.20	0.001	4.916	5.494	0.000	0.000	0.001	0.013	0.001	0.015
	15	0.10	7.00	12.00	3.50	32.52	0.001	5.018	6.544	0.000	0.000	0.001	0.014	0.002	0.016

Note: For FO and IHC, the table only lists the costs of treatments that could remove more than 10% COD in coking wastewater.

## Data Availability

Data are contained within the article and Supplementary Materials.

## Supplementary Materials

The following are available online at https://www.mdpi.com/article/10.3390/molecules29051057/s1, Table S1: Qualitative analysis of main organic compounds in coking wastewater and effluent after IHC or IHC/FO treatment. Figure S1: GC–MS analysis of raw coking wastewater (a) and effluent after IHC (b) or IHC/FO (c) treatment. Note: IHC treatments were carried out at pH 3, 40 °C, inlet pressure of 0.4 MPa, and reaction time of 60 min; IHC/FO treatments were carried out at pH 7, 40 °C, inlet pressure of 0.1 MPa, reaction time of 15 min, 12 mmol/L H_2_O_2_, and 3 mmol/L Fe(II). Figure S2: UV-Vis absorption spectra of raw coking wastewater and effluent after IHC or IHC/FO treatment. Note: experiments in (a) were carried out at 40 °C, inlet pressure of 0.4 MPa, and reaction time of 60 min; those in (b) were carried out at 40 °C, inlet pressure of 0.4 MPa, 12 mmol/L H_2_O_2_, 3 mmol/L Fe(II), and reaction time of 15 min; those in (c) were carried out at pH 3, inlet pressure of 0.4 MPa, and reaction time of 60 min; those in (d) were carried out at pH 7, inlet pressure of 0.4 MPa, 12 mmol/L HH_2_O_2_, 3 mmol/L Fe(II), and reaction time of 15 min; those in (e) were carried out at pH 3, 40 °C, and reaction time of 60 min; those in (f) were carried out at pH 7, 40 °C, 12 mmol/L H_2_O_2_, 3 mmol/L Fe(II), and reaction time of 15 min; those in (g) were carried out at pH 7, 40 °C, inlet pressure 0.1 MPa, reaction time of 15 min, and 12 mmol/L H_2_O_2_; and those in (h) were carried out at pH 7, 40 °C, inlet pressure 0.1 MPa, reaction time of 15 min, and 3 mmol/L Fe(II).

## Abbreviations

AOP	Advanced oxidation processes
HC	Hydrodynamic cavitation
IHC	Imping stream hydrodynamic cavitation
FO	Fenton oxidation
IS	Impinging stream
COD	Chemical oxygen demand
UV-Vis	Ultraviolet and visible spectrophotometry
GC–MS	Gas chromatography and mass spectrometry
UV254	Absorbance of some organic compounds in water under UV light at 254 nm wave length
Vis380	Absorbance of visible light at 380 nm
RSM	Response Surface Methodology
ANOVA	Analysis of Variance
2-D	Two-dimensional
CCD	Central composite design
C_o_	Initial concentration of pollutants
C_f_	Concentration of pollutant after treatment
OC	Operating costs
ROS	Reactive oxygen species
EPR	Electron paramagnetic resonance
3D-EEM	Three-dimensional excitation-emission matrix spectroscopy
SOA	Synchronized oxidation–adsorption
