# Supplementary material for: Treatment of Coking Wastewater Using Hydrodynamic Cavitation Coupled with Fenton Oxidation Process
Source: Molecules. 2024 Feb 28;29(5):1057. doi: 10.3390/molecules29051057 (PMC10935391; doi:10.3390/molecules29051057)
Supplement: Supplementary file 1 [file molecules-29-01057-s001.zip › molecules-2696679-supplementary.pdf]

Supplementary Materials

Treatment of Coking Wastewater Using Hydrodynamic Cavitation Coupled with Fenton Oxidation Process

Dongmei Deng [1], Ting Huang [1], Qing Li [1], Yongchun Huang [1], Yufei Sun [1,*], Jieliang Liang [2,*] and Jintian Li [2]

[1] Guangxi Key Laboratory of Green Processing of Sugar Resources, College of Biological and Chemical Engineering, Guangxi University of Science and Technology, Liuzhou 545006, China

[2] Institute of Ecological Science, Guangzhou Key Laboratory of Subtropical Biodiversity and Biomonitoring, Guangdong Provincial Key Laboratory of Biotechnology for Plant Development, School of Life Sciences, South China Normal University, Guangzhou 510631, China

* Correspondence: sunyufei@gxust.edu.cn (Y.S.); liangjl@m.scnu.edu.cn (J.L.); Tel.: +86-772-2031603 (Y.S.); +86-20-85211850 (J.L.)

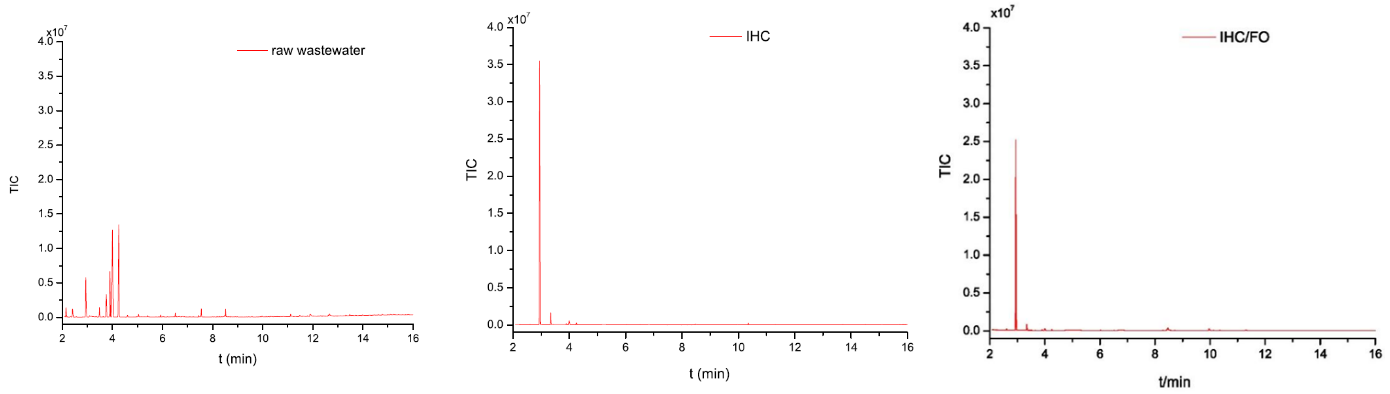

(**a**) (**b**) (**c**)

Figure S1. GC-MS analysis of raw coking wastewater (**a**) and effluent after IHC (**b**) or IHC/FO (**c**) treatment. Note: IHC treatments were carried out at pH 3, 40 °C, inlet pressure of 0.4MPa, and reaction time of 60 min; IHC/FO treatments were carried out at pH 7, 40 °C, inlet pressure of 0.1MPa, and reaction time of 15 min, 12mmol/L H_2O_2, and 3mmol/L Fe(II).

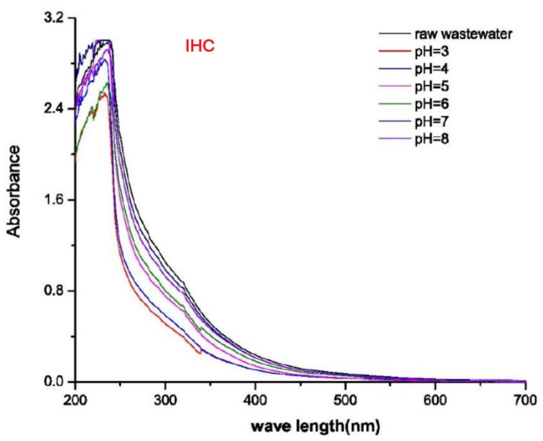
IHC
raw wastewater
pH=3
pH=4
pH=5
pH=6
pH=7
pH=8
Absorbance
wave length(nm)
0.0
0.8
1.6
2.4
3.2
200
300
400
500
600
700

(a)

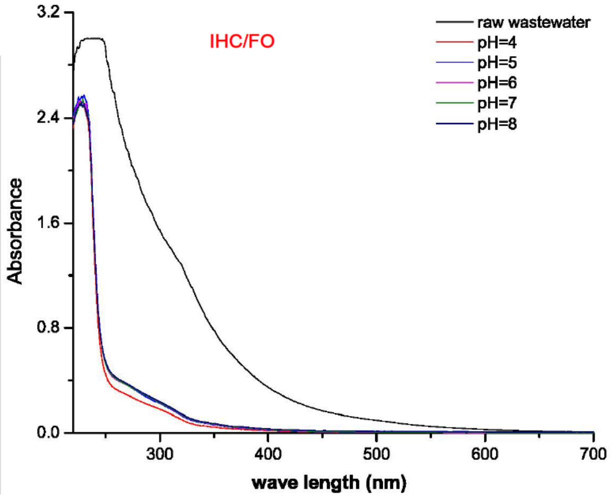
IHC/FO
raw wastewater
pH=4
pH=5
pH=6
pH=7
pH=8
Absorbance
wave length (nm)
0.0
0.8
1.6
2.4
3.2
300
400
500
600
700

(b)

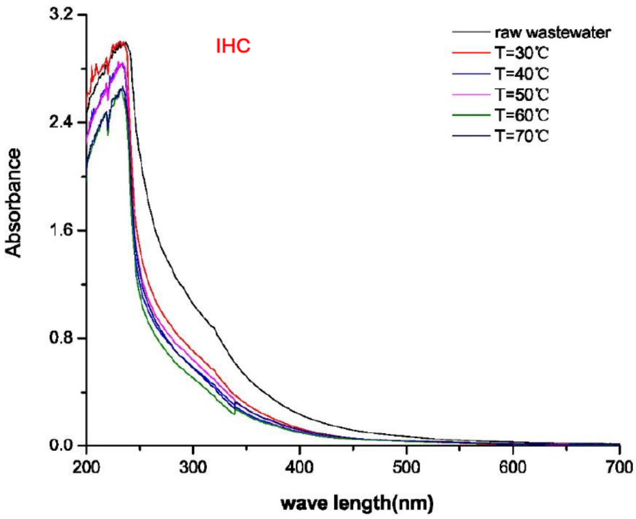
IHC
raw wastewater
T=30℃
T=40℃
T=50℃
T=60℃
T=70℃
Absorbance
wave length(nm)
0.0
0.8
1.6
2.4
3.2
200
300
400
500
600
700

(c)

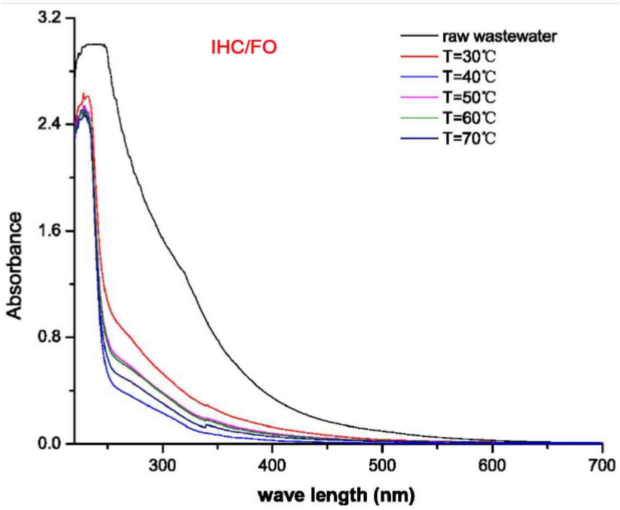
IHC/FO
raw wastewater
T=30℃
T=40℃
T=50℃
T=60℃
T=70℃
Absorbance
wave length (nm)
0.0
0.8
1.6
2.4
3.2
300
400
500
600
700

(d)

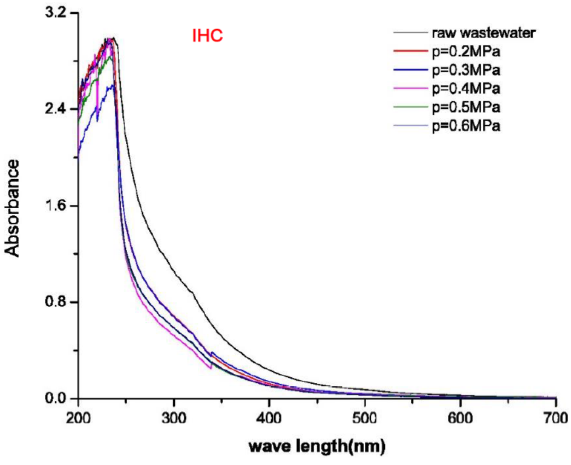
IHC
raw wastewater
p=0.2MPa
p=0.3MPa
p=0.4MPa
p=0.5MPa
p=0.6MPa
Absorbance
wave length(nm)
0.0
0.8
1.6
2.4
3.2
200
300
400
500
600
700

(e)

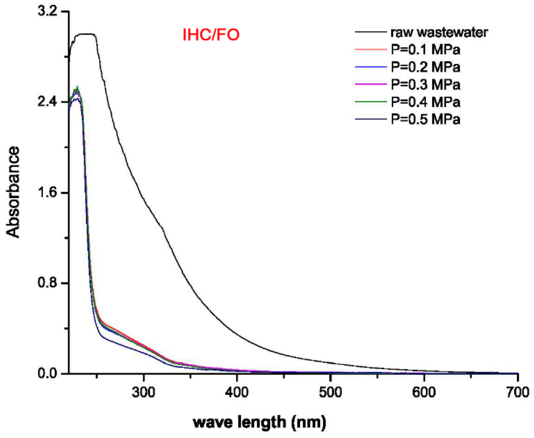
IHC/FO
raw wastewater
P=0.1 MPa
P=0.2 MPa
P=0.3 MPa
P=0.4 MPa
P=0.5 MPa
Absorbance
wave length (nm)
0.0
0.8
1.6
2.4
3.2
300
400
500
600
700

(f)

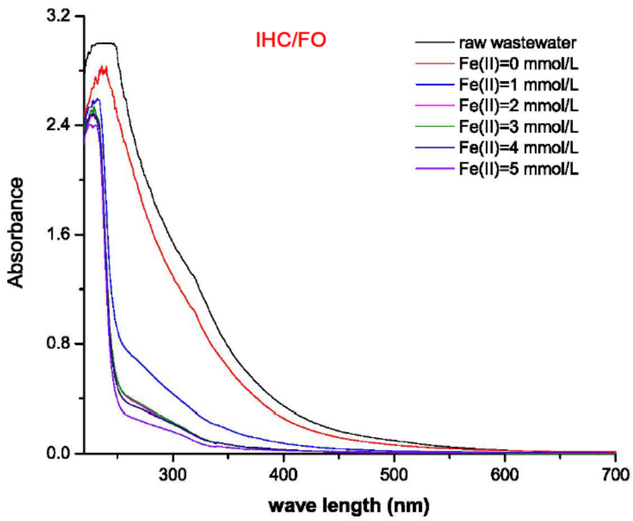

(**g**)

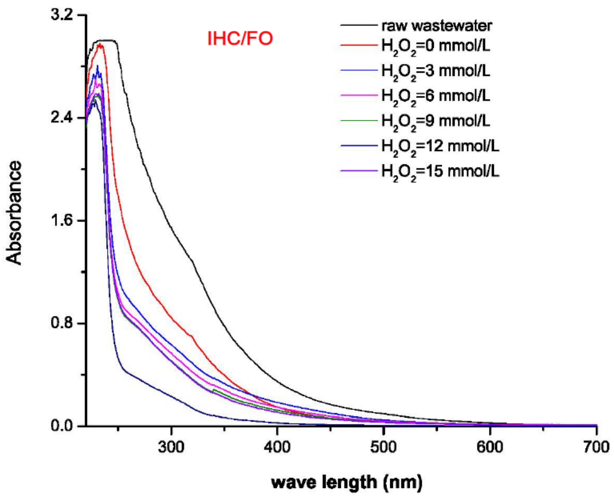

(**h**)

Figure S2. UV-Vis absorption spectra of raw coking wastewater and effluent after IHC or IHC/FO treatment. Note: (**a**) were carried out at 40 °C, inlet pressure of 0.4MPa, and reaction time of 60 min; (**b**) were carried out at 40 °C, inlet pressure of 0.4MPa, 12mmol/L H_2O_2, 3mmol/L Fe(II), reaction time of 15 min; (**c**) were carried out at pH 3, inlet pressure of 0.4MPa, and reaction time of 60 min; (**d**) were carried out at pH 7, inlet pressure of 0.4MPa, 12mmol/L H_2O_2, 3mmol/L Fe(II), and reaction time of 15 min; (**e**) were carried out at pH 3, 40°C, and reaction time 60 min; (**f**) were carried out at pH 7, 40 °C, 12mmol/L H_2O_2, 3mmol/L Fe(II), and reaction time of 15 min; (**g**) were carried out at pH 7, 40 °C, inlet pressure 0.1 MPa, reaction time 15 min and 12mmol/L H_2O_2; (**h**) were carried out at pH 7, 40 °C, inlet pressure 0.1 MPa, reaction time 15 min and 3 mmol/L Fe(II).

Table S1. Qualitative analysis of main organic compounds in coking wastewater and effluent after IHC or IHC/FO treatment.

Residence Time(min)	Organic Compound	Peak area		
		Raw Wastewater	IHC	IHC/FO
2.144	benzene	2161576	-[1]	-
2.409	1,2-dichloropropane	2199799	-	-
2.606	chloroiodomethane	-	53939	190004
2.938	toluene	959587	48052089	28993507
3.483	2-iodobutane	1586135	-	-
3.343	butyl acetate	-	1700582	799365
3.758	chlorobenzene	6198113	-	-
3.903	ethylbenzene	11289310	179694	-
4.002	1，3-dimethylbenzene	24968272	828515	393190
4.251	1,4-dimethyl-benzene	24713396	291999	-
4.604	cumene	414109	-	-
5.045	benzaldehyde	568109	-	-
5.408	2-ethyl-2-hexenal	243605	-	-
6.513	*n*-undecane	701108	-	-
7.546	*n*-dodecane	1437286	-	-
8.521	*n*-tridecane	1438405	-	-
—	others	-	-	-

1 “–”: not detected
